# 285. Outcomes and Antibiotic Use in Patients with COVID-19 Admitted to an Intensive Care Unit

**DOI:** 10.1093/ofid/ofab466.487

**Published:** 2021-12-04

**Authors:** Megan Petteys, Leigh Ann Medaris, Julie E Williamson, Travis Denmeade, Rohit Soman, William E Anderson, Michael Leonard, Christopher Polk

**Affiliations:** Atrium Health, Charlotte, North Carolina

## Abstract

**Background:**

Studies have shown the proportion of critically ill patients with COVID-19 receiving empiric antibiotics (ABX) greatly exceeds those with culture-proven bacterial co-infections. However, the benefits of continuing ABX in culture-negative (CxN) cases is unknown; this practice may increase the risks associated with ABX overuse. The purpose of this study was to evaluate outcomes and antibiotic use (AU) in intensive care unit (ICU) patients with COVID-19 based on culture results.

**Methods:**

This was a multicenter, retrospective cohort study evaluating adults in an ICU for the first episode of ABX initiated following a confirmed COVID-19 diagnosis between September to December 2020. Blood and/or respiratory cultures must have been obtained within 24 hours (h) of ABX initiation. Patients were categorized into three groups: 1) CxN, ABX discontinued ≤ 72 h, 2) CxN, ABX continued > 72 h, or 3) Culture-positive (CxP). Data on AU was obtained from electronic medication administration records. The primary outcome was clinical success, defined as being discharged alive or > 2-point decrease in the World Health Organization Clinical Progression Scale score from day of ABX initiation to day 30.

**Results:**

A total of 65 patients were included with 35.4% being CxP. ABX were discontinued ≤ 72 h in 23.8% of CxN patients. Methicillin-susceptible *Staphylococcus aureus* was the most common organism in 52.2% of CxP patients (66.7% respiratory; 16.7% blood; 16.7% both). Anti-methicillin-resistant *Staphylococcus aureus* and anti-pseudomonal antibiotics were the most prescribed for the initial regimen (Table 1). ABX de-escalation occurred in 58.5% of patients. Initial ABX duration was significantly longer in the CxP group (P < 0.01). No significant difference in clinical success was observed (Table 2). Although not significantly different, the highest rate of adverse events occurred in the CxN and ABX continued > 72 h group (40.6%).

Table 1. Antibiotic Use in ICU Patients with COVID-19

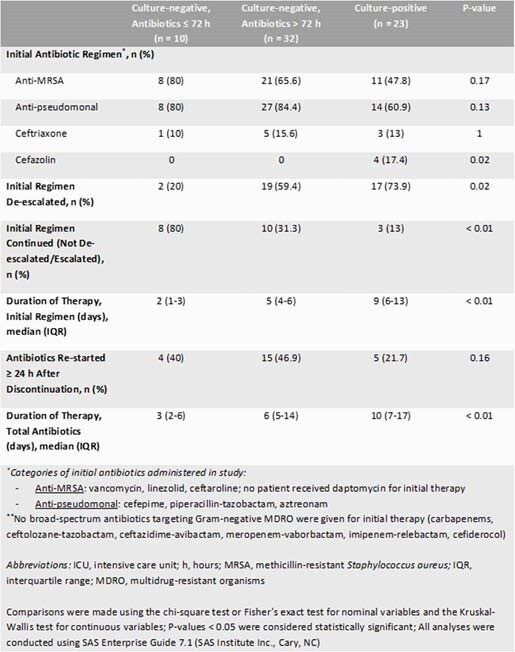

Table 2. Clinical Outcomes and Adverse Events in ICU Patients with COVID-19

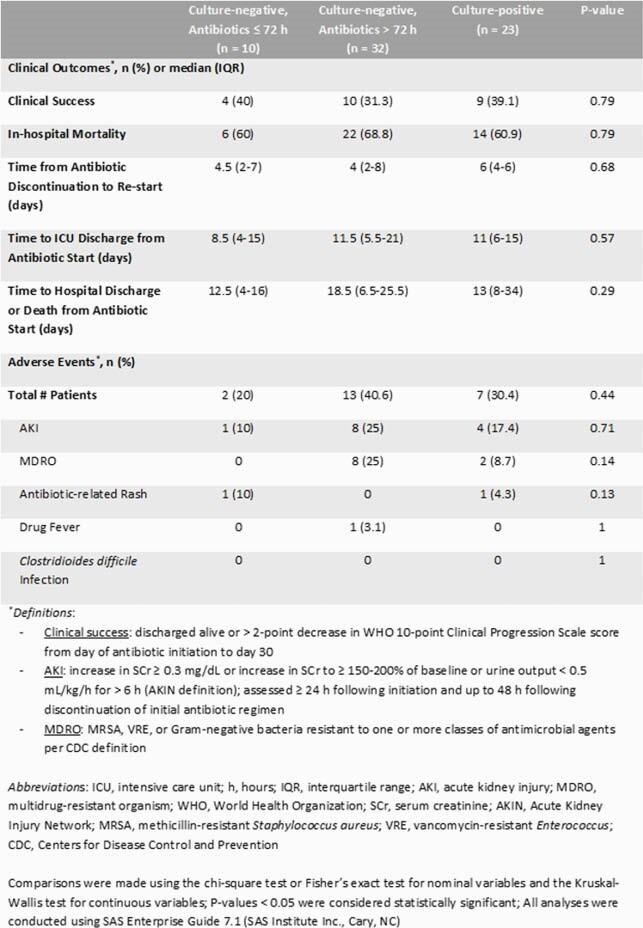

**Conclusion:**

In ICU patients with COVID-19, empiric broad-spectrum ABX are often overutilized with an inertia to de-escalate despite negative culture results, potentially increasing the risk of adverse events. This remains an important area for focused antimicrobial stewardship efforts to mitigate the development of multidrug resistance.

**Disclosures:**

**Christopher Polk, MD**, **Atea** (Research Grant or Support)**Gilead** (Advisor or Review Panel member, Research Grant or Support)**Humanigen** (Research Grant or Support)**Regeneron** (Research Grant or Support)

